# A comprehensive and quantitative SEM–EDS analytical process applied to lithium-ion battery electrodes

**DOI:** 10.1038/s41598-025-89362-w

**Published:** 2025-02-13

**Authors:** Teruki Kato, Kunihiro Goto, Takahiro Niwa, Tsukasa Shimizu, Akinobu Fujii, Bunyo Okumura, Hideaki Oka, Hiroaki Kadoura

**Affiliations:** Toyota Central R&D Labs., Inc., 41-1, Yokomichi, Nagakute, Aichi 480-1192 Japan

**Keywords:** Characterization and analytical techniques, Computational methods

## Abstract

The combination of scanning electron microscopy (SEM) images and energy-dispersive X-ray spectroscopy (EDS) maps (SEM–EDS analysis) enables the analysis of the relationship between the microstructures and elemental compositions of the surfaces of materials. However, conventional SEM–EDS analyses lack comprehensiveness and quantitativeness, resulting in potential inaccuracies in reflecting the properties of the entire sample and variations in the results depending on the analyst. Therefore, herein, we propose an objective SEM–EDS analytical process that addresses the aforementioned issues. Comprehensiveness was addressed by acquiring large volumes of SEM images through automated capturing, whereas quantitativeness was addressed through microstructural analysis of the SEM images based on image features, model-based dimension reduction and clustering methods, and similarity analysis of the elemental distribution in EDS maps based on statistical distances. The proposed method was used to analyze the degradation of lithium-ion battery electrodes, affording objective results that align with subjective insights into the changes in the morphology and composition of solid electrolyte interphase (SEI) films accompanying degradation.

## Introduction

In materials science, it is essential to conduct detailed analyses of the surface characteristics of materials to develop high-performance materials. This is indispensable in the development of advanced materials such as batteries, semiconductors, and catalytic materials, where the microstructure of the surface and composition of the elements constituting the material significantly affect its performance. In battery materials, minor changes to the surface of the electrode particles influence the chemical reactions at the interfaces, affecting the battery lifespan and charge–discharge efficiency. Therefore, accurate analyses of the structure and chemical composition of the surface of battery materials are vital for improving battery performance^1,2^. For semiconductor materials, minor surface defects or impurities can hinder electron movement and degrade device performance. Hence, a detailed understanding of the surface conditions of semiconductor materials is important for enhancing the reliability and efficiency of electronic devices^3,4^. In catalytic materials, the number, arrangement, and composition of active sites on the surface of the material determine the activity and selectivity of the catalyst. Thus, determining the structure and chemical composition of a catalyst surface is important for optimizing catalytic reactions and developing high-performance catalytic processes^5^. The combination of high-resolution observations of the surface structures with analysis of the surface composition can maximize material performance. Standard methods for the detailed observation of microstructures on material surfaces involve SEM, including secondary and backscattered electron images. To reveal the chemical composition of a material’s surface, EDS is used to obtain element maps (EDS maps). The combination of SEM images and EDS maps is called SEM–EDS analysis and can reveal the relationship between the microstructure and composition of the surfaces of materials^6^. Previously, standard SEM–EDS analysis was subjective, based on the manual observation of a small amount of measured data, which lacked comprehensiveness and quantitativeness. Comprehensiveness refers to the use of a large amount of data across a broad area of the sample, while quantitativeness refers to the quantification of the differences in properties, such as physical values and surface structures. Analyses using limited data may not reflect the overall properties of the sample, whereas qualitative analyses may yield different results depending on the analyst. Therefore, it is essential to develop an SEM–EDS analytical method that is both comprehensive and quantitative to objectively analyze the relationship between the surface structure and composition. This is referred to as objective SEM–EDS analysis and is the main subject of this study. Challenges in achieving objective SEM–EDS analysis include the comprehensive acquisition of large datasets and the development of quantitative analytical methods that combine SEM images and EDS maps.

Recent studies related to resolving the comprehensiveness challenge in the field of materials science include deep learning-based classification using a large number of SEM images^7,8^, segmentation^9–12^, particle analysis^13^, and image restoration^14^. In biology, integrated analytical processes that combine automatic capturing, stitching, viewing, and analysis of SEM images have been proposed^15–17^. However, these methods aim solely at analyzing microstructures based on SEM images and cannot be combined with compositional information. Studies focusing on resolving the quantitativeness challenge in SEM–EDS analyses have included quantitative analytical methods based on superpixel segmentation of SEM–EDS maps and clustering of the compositional ratios for phase identification in cementitious materials^18–22^. In addition, quantitative analytical methods based on elemental correlation segmentation of SEM–EDS maps for phase identification in soil aggregates have been reported^23^. However, these methods aim at separating phases using both microstructural information from SEM images and compositional information from EDS maps and are not applicable for analyzing the relationship between microstructure and composition, which is the main topic of this study. Additionally, data analysis methods such as dimension reduction and clustering in these studies are model-free, designed for the analysis of small datasets, and require the simultaneous insertion of all target data into the algorithm. Therefore, their application to large datasets requires substantial computational resources and time. Thus, a comprehensive and quantitative SEM–EDS analytical method for analyzing the relationship between surface structure and composition has not been established yet.

In this study, we propose an objective SEM–EDS analytical process that combines three steps: (Step A) creation of an SEM–EDS patch image dataset based on a comprehensive automatic capture of SEM images; (Step B) quantitative analysis of microstructures based on the SEM image features; and (Step C) quantitative analysis of elemental distribution based on EDS maps. During dataset creation (Step A), the SEM images were automatically captured as described by Kume et al*.*^15,16^ and Son et al*.*^17^. This allowed the facile acquisition of comprehensive SEM image data, enabling analysis with less arbitrariness in the observation area. In the microstructural analysis (Step B), the features were calculated for each patched SEM image, followed by dimension reduction and clustering. Analyses based on textural features^24,25^ can quantitatively distinguish the differences in microstructures that are visually recognized. Furthermore, model-based dimensional reduction and clustering facilitate the computation of large amounts of data. During elemental distribution analysis (Step C), the differences in the elemental distribution for each cluster of microstructures were analyzed using the statistical distance^26^. The quantification of the distance between elemental distributions allows the quantification of the overall trends that are not reflected by the mean or variance alone. Through these steps, it is possible to quantitatively determine the differences in elemental distribution for regions with quantitatively distinguished microstructures using comprehensive data.

The proposed analytical process was used to analyze the degradation of lithium-ion battery electrodes. In lithium-ion battery graphite electrodes, a SEI film is formed during initial charging and discharging, consisting of compounds such as lithium fluoride and lithium carbonate^27–29^. Additionally, capacity degradation occurs when lithium-ion batteries are used at high temperatures, which is caused by lithium deactivation associated with changes in the morphology and composition of the SEI film on the graphite electrode surface^30,31^. These results are based on theoretical chemical analyses or subjective analyses through visual observation. Recent research on the quantitative analysis of microstructural data of battery materials^32^ involved segmentation by deep learning^33–36^, super-resolution^37^, and image quality evaluation^38^. However, these methods do not utilize large automatically-measured datasets in their analytical process, nor do they combine analyses of surface structures with compositional information. In our study, we applied the proposed method to multiple lithium-ion battery electrodes with varying degrees of degradation, enabling a comprehensive and quantitative analysis of the relationship between surface structure and composition. Our analytical method yielded results that are consistent with the findings of traditional subjective analyses. Furthermore, we analyzed the detected blobs (circular regions) in the SEM images that are considered related to the morphologically changed SEI films due to degradation. The combination of the microstructure results with the blob results verified that the proposed method, particularly when analyzing lithium-ion battery electrodes, captured the differences in blob features related to the SEI film.

In summary, herein, we propose an objective and concretized SEM–EDS analytical process by combining appropriate data measurements and analytical methods. Furthermore, the proposed method was applied to the analysis of the degradation of lithium-ion battery electrodes, exhibiting consistent results with the traditional qualitative insights reported in the literature for electrode degradation.

## Results and discussion

### Proposed analytical process

**Fig. 1:**
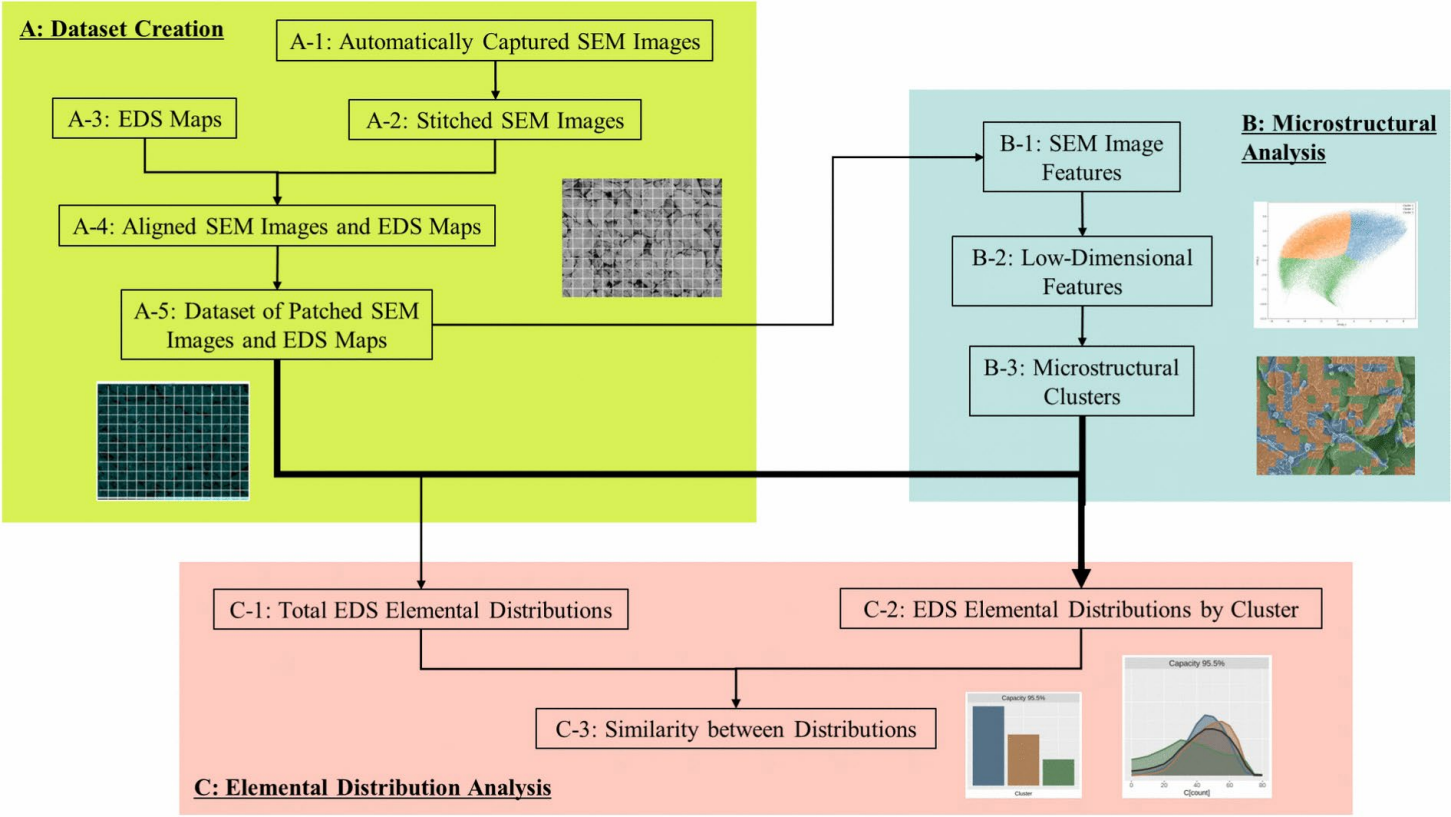
Overview of the proposed objective SEM–EDS analytical process.

Figure 1 presents the objective SEM–EDS analytical process proposed in this study. Initially, in Step A (dataset creation), a dataset of aligned SEM–EDS patch images was created by acquiring SEM images through automatic capturing, stitching of SEM images, measurement of EDS maps, alignment of SEM images with EDS maps, and patch division. In Step B (microstructural analysis), the dataset of the patched SEM images was used to cluster the microstructures. This includes extracting the SEM image features, reducing the dimensionality of the features, and clustering low-dimensional features. In Step C (elemental distribution analysis), an integrated analysis of the microstructure clustering results and elemental distribution in the patched EDS maps was conducted. For each microstructural cluster, the elemental distribution was created from the corresponding patches of the EDS maps and their similarity to the overall elemental distribution was calculated. By comparing the similarities obtained for each cluster, it is possible to quantitatively understand the contribution of the elemental distribution in areas with different microstructural features to the overall trend of the elemental distribution. The specific technologies used in each step are described in the Methods section.

### Measurement results

**Fig. 2:**
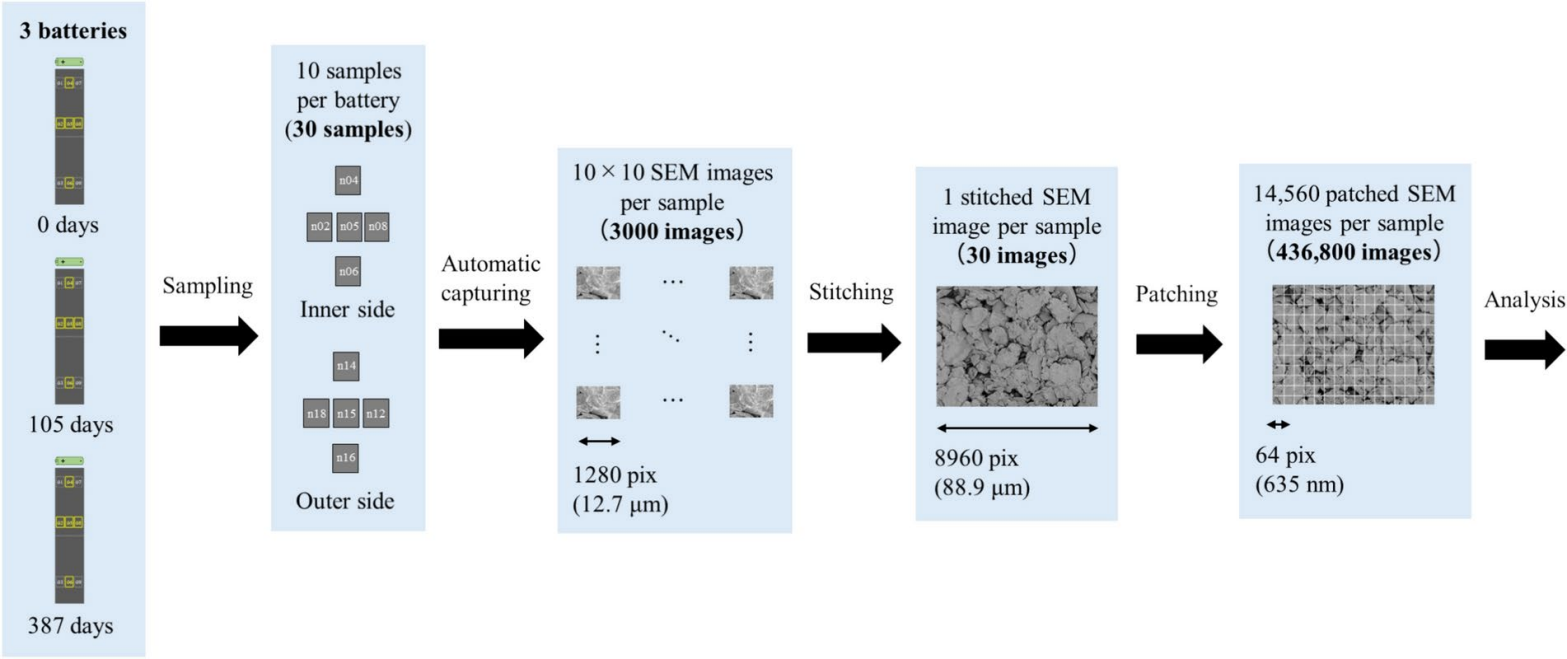
Overview of the SEM image dataset used in this study. The dataset consists of three batteries with different storage durations: initial (0 d), stored for 105 d, and stored for 387 d. For each battery, 10 samples were taken from the electrodes, as described in the Methods section and shown in Fig. 12. SEM images were captured from each sample to generate a comprehensive dataset for analysis.

**Fig. 3:**
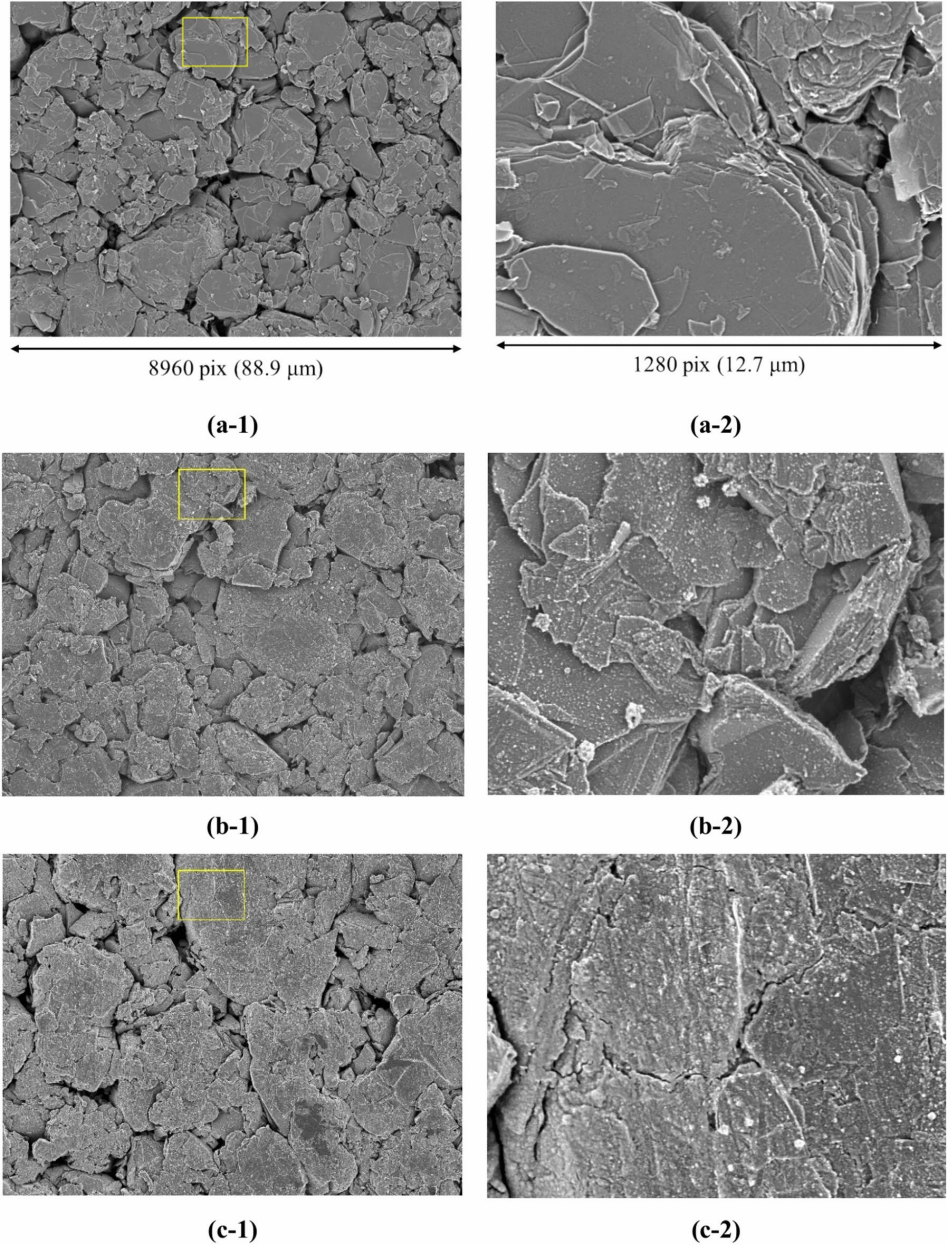
Stitched and magnified views of automatically captured SEM images. (a-1), (b-1), and (c-1) represent examples of stitched SEM images of lithium-ion battery electrode samples with capacity retention rates of 100%, 95.5%, and 85.2%, respectively. (a-2), (b-2), and (c-2) are images of the areas outlined in yellow in (a-1), (b-1), and (c-1), respectively, indicating that the physical size per pixel is the same in both the original images and the magnified images.

**Fig. 4:**
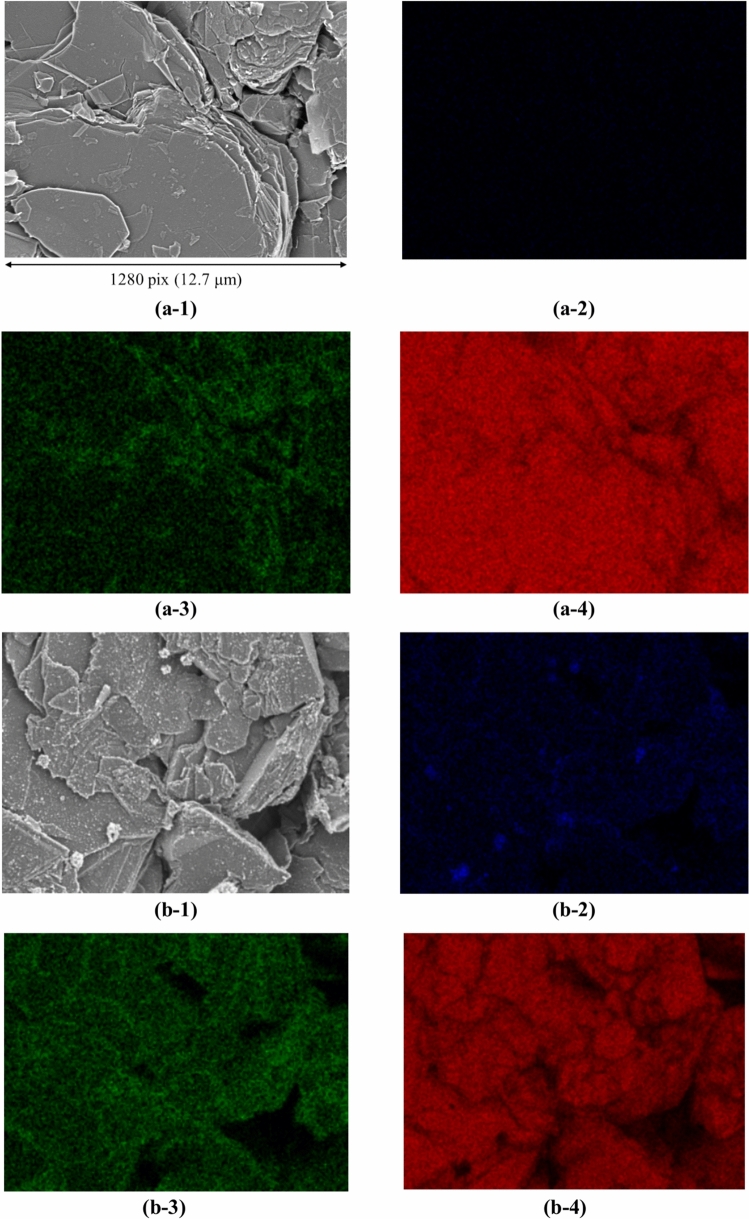

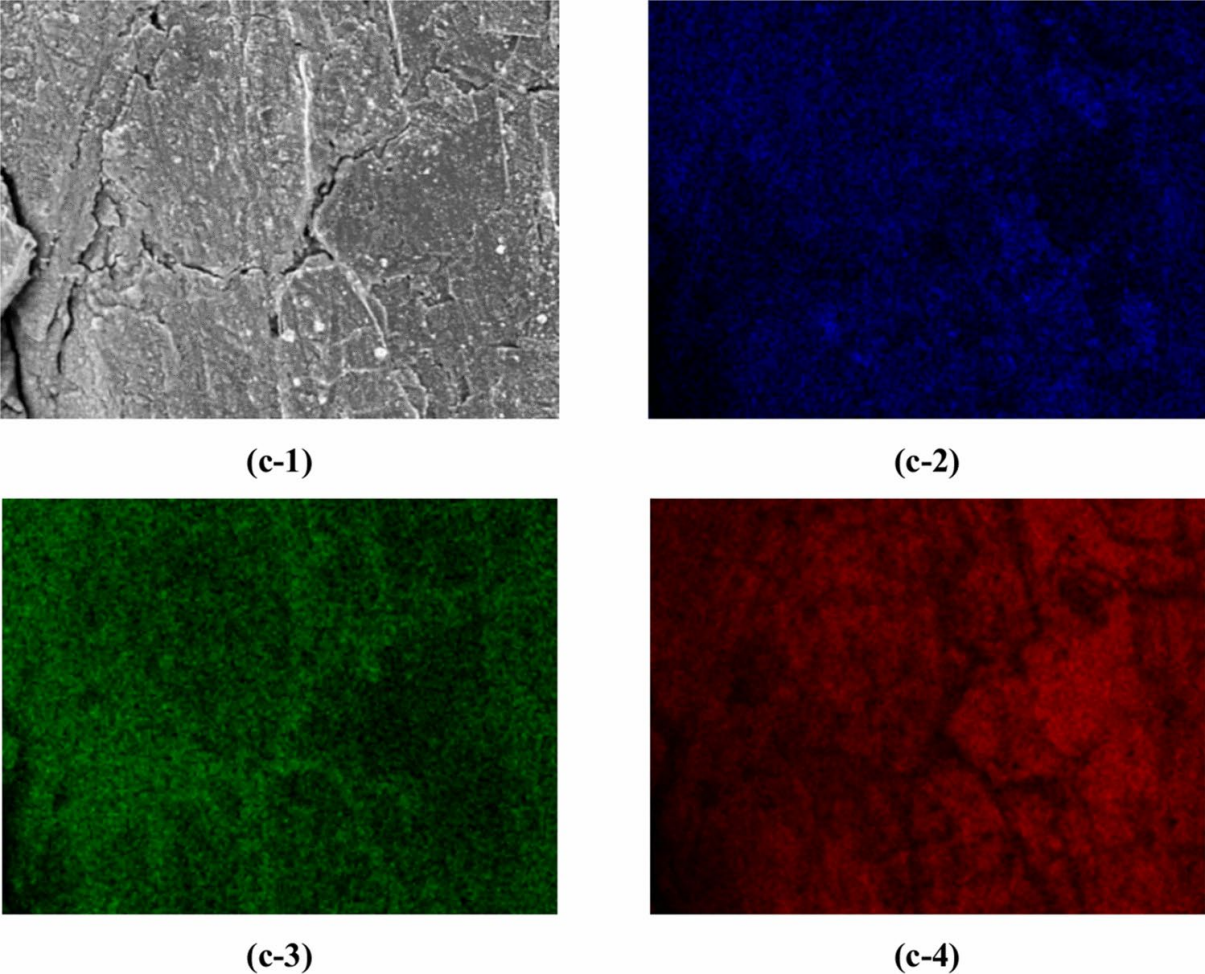
Elemental distribution maps (EDS maps) of F, O, and C corresponding to the same fields of view as the SEM images in Fig. 3. Panels (a-1), (b-1), and (c-1) show the SEM images from Fig. 3 for a new battery sample (100% capacity retention), a degraded battery (95.5% capacity retention), and a further degraded battery (85.2% capacity retention), respectively. Panels (a-2), (b-2), and (c-2) show the elemental distribution of fluorine (F) for the corresponding samples. Panels (a-3), (b-3), and (c-3) show the elemental distribution of oxygen (O), and panels (a-4), (b-4), and (c-4) show the elemental distribution of carbon (C) for the corresponding samples.

This section presents the measurement results obtained from both SEM images and EDS maps. Figure 2 shows the SEM image dataset used for microstructural analysis. In this study, three lithium-ion batteries with different degrees of degradation were analyzed by collecting ten samples from each battery, resulting in a total of thirty samples. The batteries had capacity retention rates of 100%, 95.5%, and 85.2%, indicating one new battery and two degraded batteries. SEM images and EDS maps were obtained for all samples. Details of the samples are included in the Methods section. In Step A, a dataset consisting of paired patch images of SEM images and EDS maps (F, O, and C) was created, totaling 436,800 pairs. For each sample, 100 SEM images (backscattered electron images) were automatically captured in a grid pattern of 10 × 10, requiring approximately 1.5 h per sample. Thus, the total time required for automatic capture of the images of the 30 samples was approximately 45 h. Figure 3 presents examples of the stitched 100 SEM images and their magnified views. It is evident from Fig. 3 that high-magnification SEM images across a broad area can be obtained through automatic capture. Additionally, Fig. 4 (EDS maps) shows the elemental maps of F, O, and C corresponding to the same fields of view as the SEM images. These maps enable a detailed examination of the elemental distributions, highlighting the differences in the elemental concentrations among the regions of interest. Comparison of the SEM images of the new battery (Fig. 3a) with those of the degraded batteries (Fig. 3b and c) revealed the presence of SEI films in the degraded batteries, indicating a morphological change due to degradation. This morphological change is further corroborated by the increased presence of fluorine and oxygen in the EDS maps of the degraded batteries.

### Microstructural analysis results

**Fig. 5:**
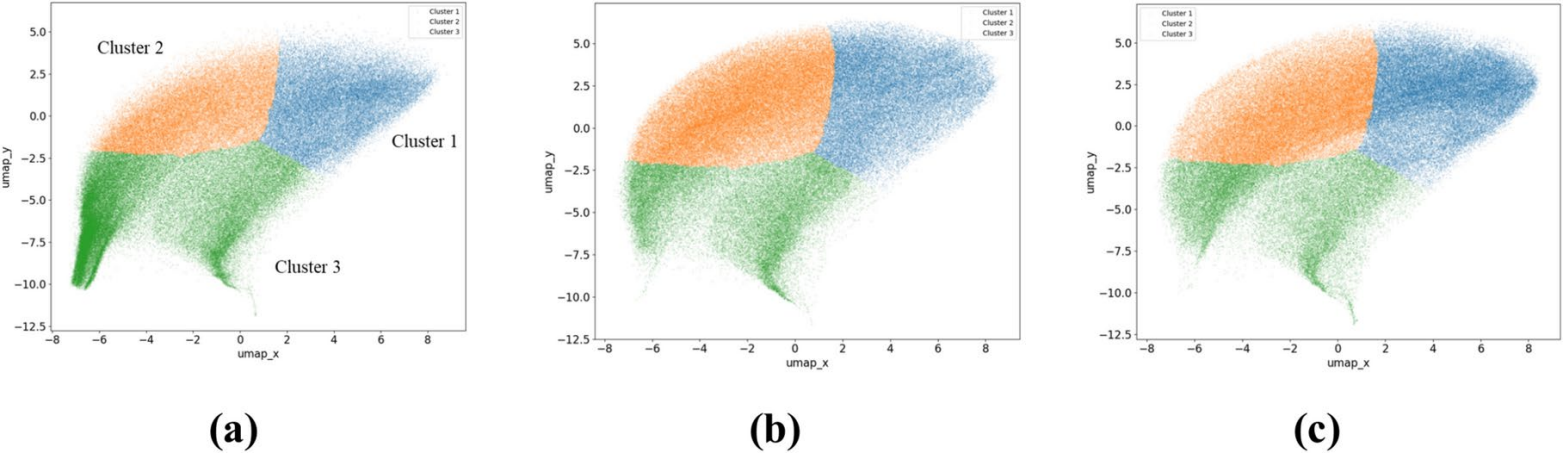
Scatter plots of clustered SEM image features. (**a**), (**b**), and (**c**) represent the SEM image data results of lithium-ion battery electrodes with capacity retention rates of 100%, 95.5%, and 85.2%, respectively. Blue, orange, and green represent clusters 1, 2, and 3, respectively.

**Fig. 6:**
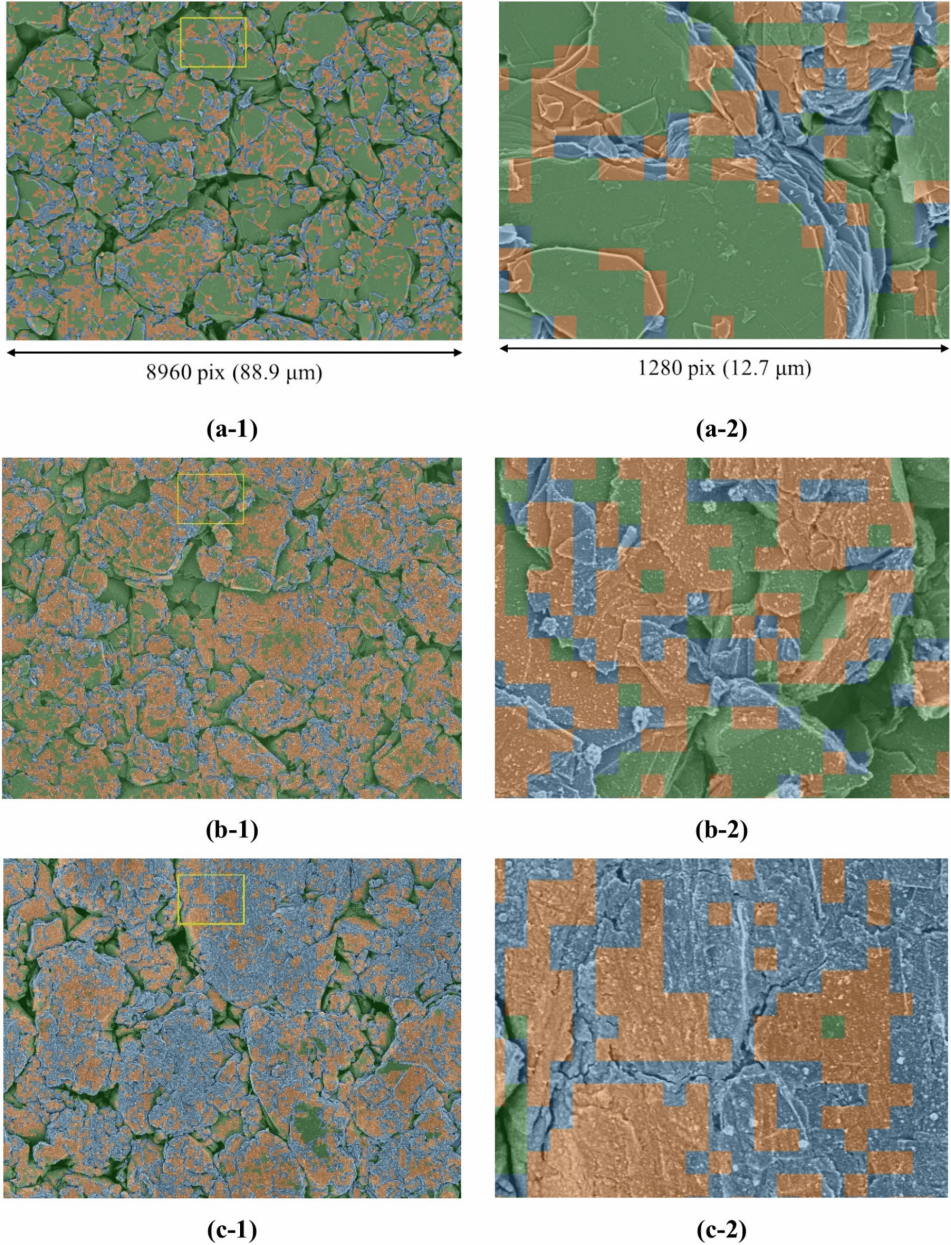
Overlaid images of microstructure clustering results on SEM images. (a-1), (b-1), and (c-1) represent examples of SEM image data of lithium-ion battery electrodes with capacity retention rates of 100%, 95.5%, and 85.2%, respectively, and show the same fields of view as in Fig. 3. (a-2), (b-2), and (c-2) show the magnified views of the areas within the yellow frames in (a-1), (b-1), and (c-1), respectively. Blue, orange, and green represent clusters 1, 2, and 3, respectively. The square segments visible in the images represent the individual patches used for the analysis, with each patch covering an area of 635 × 635 nm.

In Step B, the features were extracted from the SEM patch images in the dataset, followed by reduction to two dimensions and clustering. Figure 5 shows the results of clustering the low-dimensional features, with the number of clusters set to three. The method for determining the number of clusters is described in the Methods section and Supplementary Information. It is evident from Fig. 5 that in the results of the degraded batteries (b) and (c), the areas of clusters 1 and 2 have expanded, whereas the area of cluster 3 has shrunk compared to the result of the new battery (a). Images overlaying the clustering results on the SEM images in Fig. 3 are shown in Fig. 6. The magnified views in Fig. 6 show that clusters 1 and 2 correspond to areas with coarse microstructural features, whereas cluster 3 corresponds to areas with smooth microstructural features or gaps between particles. Furthermore, in the results of the degraded batteries in Fig. 6b and c, comparison of the areas of clusters 1 and 2 indicates that the particles presumed to have morphologically changed due to degradation are mostly found in cluster 1. This confirms that clustering-based analysis can effectively distinguish the differences in the microstructural features of the SEM image.

#### Blob analysis related to SEI films

**Fig. 7:**
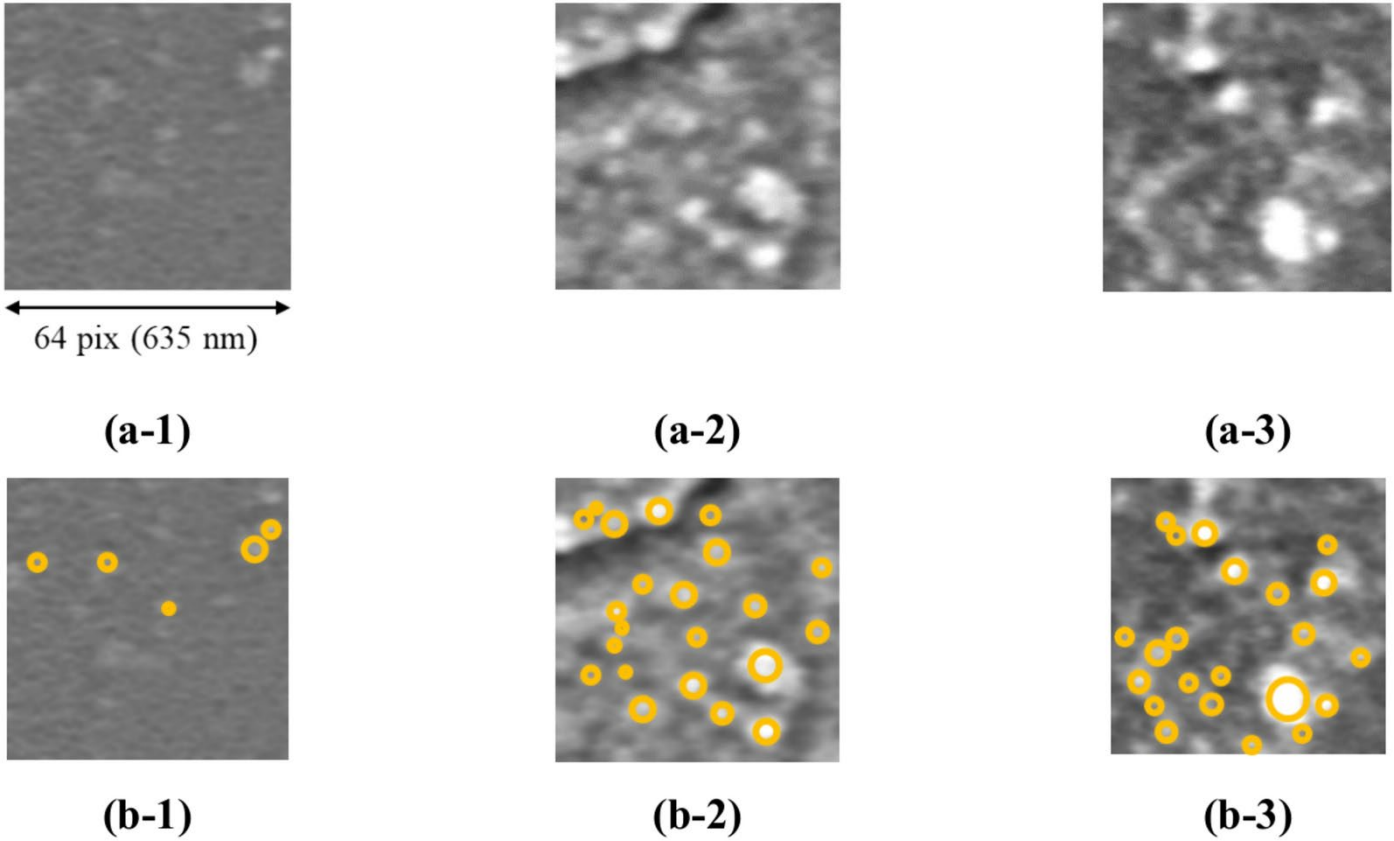
Blob detection results. Examples of blob detection results on SEM patch images using the DoG filter. (a-1), (a-2), and (a-3) represent SEM patch images of lithium-ion battery electrodes with capacity retention rates of 100%, 95.5%, and 85.2%, respectively. (b-1), (b-2), and (b-3) show the results of the blob detection for the SEM images (a-1), (a-2), and (a-3), respectively.

**Fig. 8:**
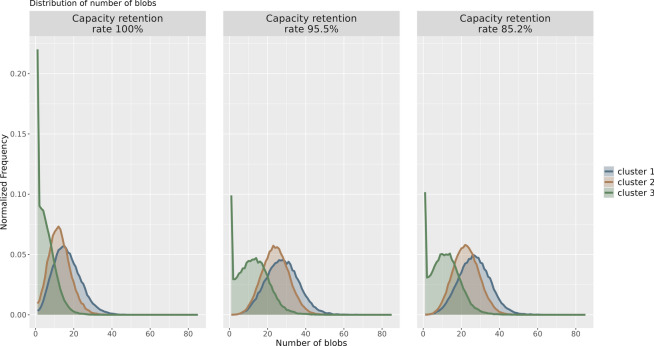
Frequency distribution of blobs detected using the DoG filter. Visualized results of the frequency distribution of the number of blobs detected from the patched SEM images of lithium-ion battery electrode samples with different capacity retention rates and by microstructure cluster.

Microstructural analysis suggested that the differences between clusters 1 and 2 were related to the differences in the SEI films that changed morphologically due to degradation. An analysis was conducted to detect blobs (circular areas) in the SEM images, which are presumed to be related to the SEI films, and their relationship with the microstructural analysis results was examined. SEI films appear in SEM images as circular areas with different brightness values (blobs), therefore, it is expected that they can be detected using the standard difference of Gaussian (DoG) filter^39^. An example of the blob detection results using the DoG filter on patched SEM images binarized using Otsu’s method^25^ is shown in Fig. 7. It can be confirmed from Fig. 7 that the blobs related to the SEI films can be detected satisfactorily using the DoG filter. This blob-detection process was conducted on all patched SEM images in the dataset, and the number of blobs detected in each patch was counted. Furthermore, for each of the aforementioned microstructure clusters, a graph of the normalized frequency distribution of the number of blobs in the corresponding patches is shown in Fig. 8. Regardless of the capacity of the batteries, the number of blobs increased in the order of clusters 3, 2, and 1 (Fig. 8). This result is consistent with the intuitive interpretation mentioned in Fig. 6, where SEI films with morphological changes due to degradation were more prevalent in the order of clusters 3, 2, and 1. This confirms that when the proposed method is applied to lithium-ion battery electrodes it can capture the differences in the blob features related to the SEI film.

### Elemental distribution analysis results

**Fig. 9:**
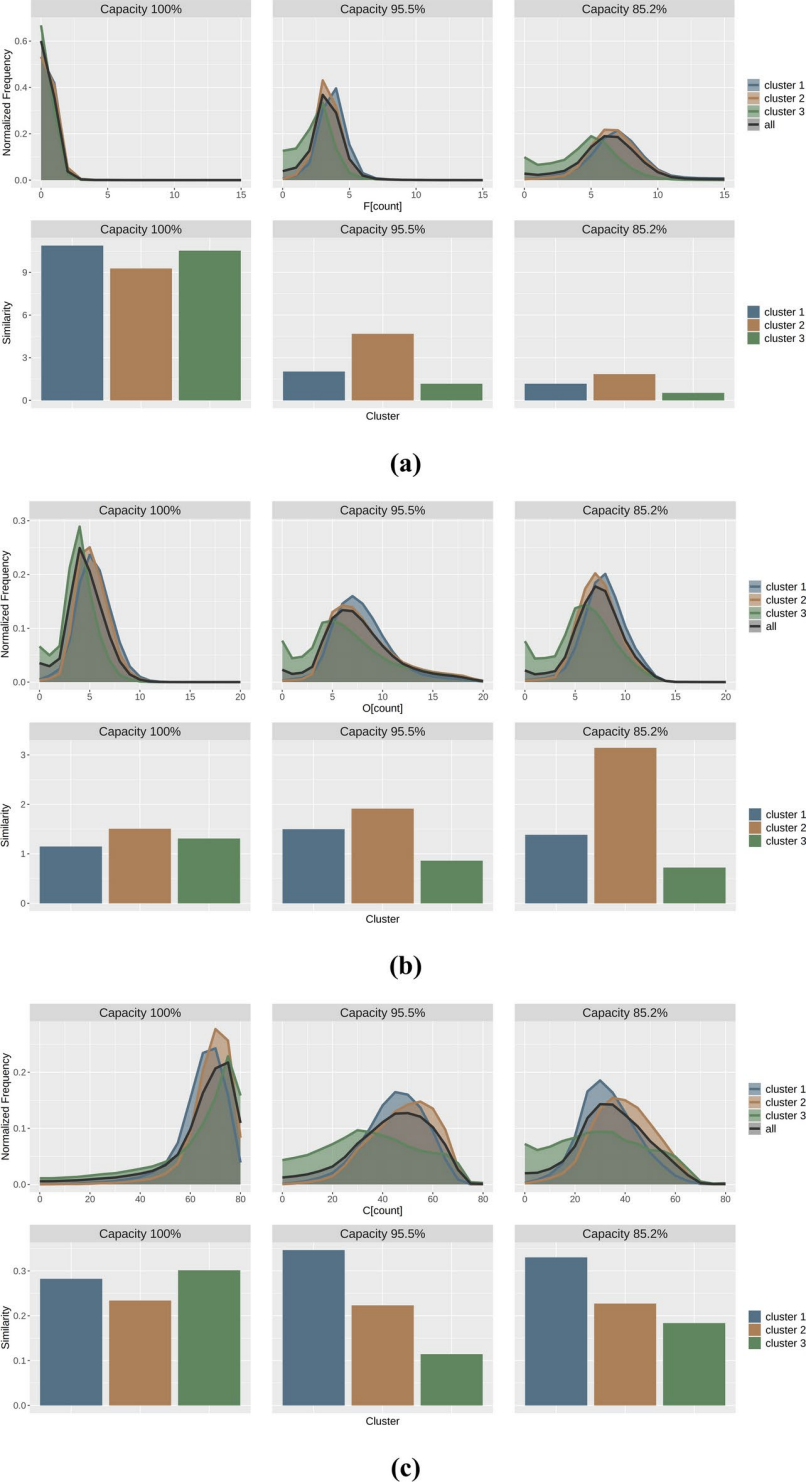
Elemental distribution analysis results. (**a**), (**b**), and (**c**) present the elemental distributions of F, O, and C, respectively. The upper section shows the elemental distribution calculated for each battery with different capacity retention rates and for each microstructure cluster. The lower section shows the similarities between the overall elemental distribution and the elemental distribution for each cluster.

For each battery with different capacity retention rates and for each microstructure cluster, the corresponding patched EDS maps (F, O, and C) were extracted, and their mean brightness values were calculated. A graph normalizing the frequency distribution of the calculated average elemental amounts for each patch is shown in the upper section of Fig. 9. Furthermore, the results of calculating the similarities between the overall elemental distribution and the elemental distribution for each cluster are shown in the lower section of Fig. 9. From the elemental distribution of each battery in the upper section of Fig. 9, it can be observed that upon degradation (decrease in the capacity retention rate), the amounts of fluorine and oxygen increase, while the amount of carbon decreases. Moreover, from the similarities between the distributions in the lower section, it is noted that for the new battery, the differences in similarities between clusters are small; however, for the degraded batteries, the similarities are greater in clusters 1 and 2 than in cluster 3. This indicates that the regions corresponding to clusters 1 and 2, which had coarse microstructural features, contribute more to the overall trend of the elemental distribution, whereas the contribution was smaller in the region of cluster 3, which corresponds to smooth microstructures or gaps. Comparison of the elemental distributions of clusters 1 and 2 in the two degraded batteries revealed that for elements F and O, cluster 1 had a higher elemental amount, deviating from the overall trend, whereas for C, cluster 1 had a lower elemental amount, resembling the overall trend.

## Discussion

The comprehensiveness and quantitativeness of the proposed method are discussed based on the measurement and analytical results.

### Comprehensiveness

The total number of high-magnification SEM images obtained through automatic capture was 3000, while the number after patching was 436,800. Figure 2 presents the comprehensive nature of the dataset, showing the large field of view captured through the stitching of 100 SEM images for a single sample. This approach allows for the analysis of extensive regions while maintaining a high resolution. Compared with related studies on SEM–EDS data analysis^18,23^ involving 19 and 18 SEM images, our data number is at least 100 times higher. The ability to handle such large datasets is critical for ensuring that the analysis reflects the overall properties of the sample and minimizes the risk of bias introduced by limited data selection. Although it is conceivable that an expansion to even more data may be necessary depending on the analysis subject, our analytical process was designed to handle large-scale data for both measurement and analysis, making the expansion straightforward.

### Quantitativeness

The microstructural results revealed that clusters 1 and 2 corresponded to areas with coarse surface structures and a high number of blob features related to SEI films, whereas cluster 3 corresponded to areas with smooth surface structures or gaps and fewer blob features. Furthermore, the elemental distribution analysis results confirmed that in the degraded batteries, clusters 1 and 2 had higher amounts of fluorine and oxygen and lower amounts of carbon. These quantitative analysis results are consistent with existing qualitative insights into electrode degradation phenomena, where SEI films composed of lithium fluoride and lithium carbonate change morphologically and cover the carbon substrate. Thus, the proposed method demonstrated excellent quantitative analysis results (i.e., quantification of qualitative knowledge), which aligned with traditional analysis-based qualitative reports.

## Methods

As shown in Fig. 1, the proposed analytical process involves three steps: dataset creation, microstructural analysis, and elemental distribution analysis. The details of each step are as follows:

### Step A: dataset creation

#### Step A-1: automatic capturing of SEM images

**Fig. 10:**
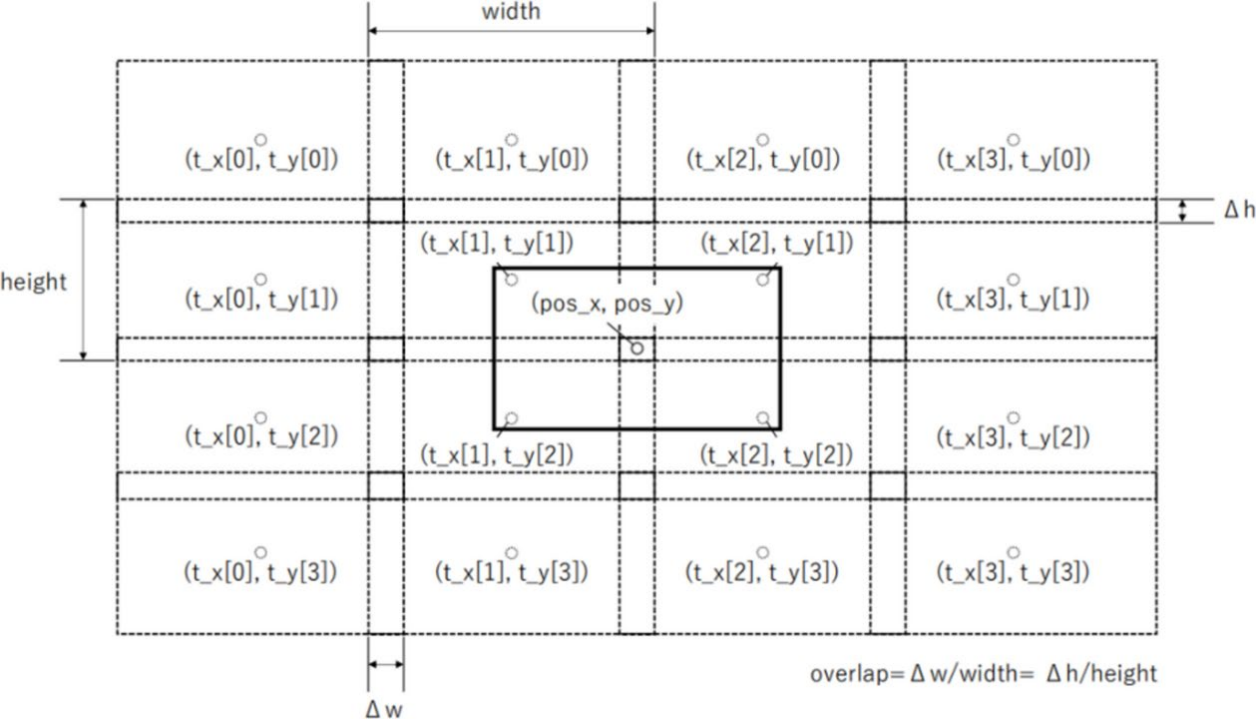
Coordinates for automatic capturing.

For a comprehensive microstructural analysis using SEM images, high-magnification SEM images were acquired through automatic capturing, similar to the method reported by Kume et al*.*^15,16^ and Son et al*.*^17^. Initially, a grid of capturing coordinates is created based on the capturing center coordinates, size per capture, number of captures, and overlap rate (Fig. 10). Considering subsequent stitching, the coordinates are determined in a way that the edges of the adjacent SEM images overlap based on the overlap rate. Then, using the communication function of the SEM instrument, continuous field capturing was performed by sequentially transmitting the coordinates.

#### Step A-2: stitching of SEM images

The obtained grids of the SEM images are stitched vertically and horizontally to create high-magnification SEM images covering a wide area. However, simple stitching can result in discontinuous images at the borders because of stage position shifts and image distortions during the continuous field capture of the SEM images^14,40–43^. Therefore, in this study, stitched images with continuous borders were created by stitching the SEM images, including the overlaps, using the grid/collection stitching function of PyImageJ^44^, as reported by Kume et al*.*^15,16^ and Son et al*.*^17^.

#### Step A-3: measurement of EDS maps

A single low-magnification EDS map was used to cover the area captured using the automated SEM image capturing. EDS maps were created by applying region of interest (ROI) techniques to visualize the X-ray count values corresponding to specific elements. Additionally, considering the subsequent alignment process, a low-magnification SEM image was captured in the same field of view as that of the EDS measurement.

#### Step A-4: alignment of SEM images and EDS maps

**Fig. 11:**
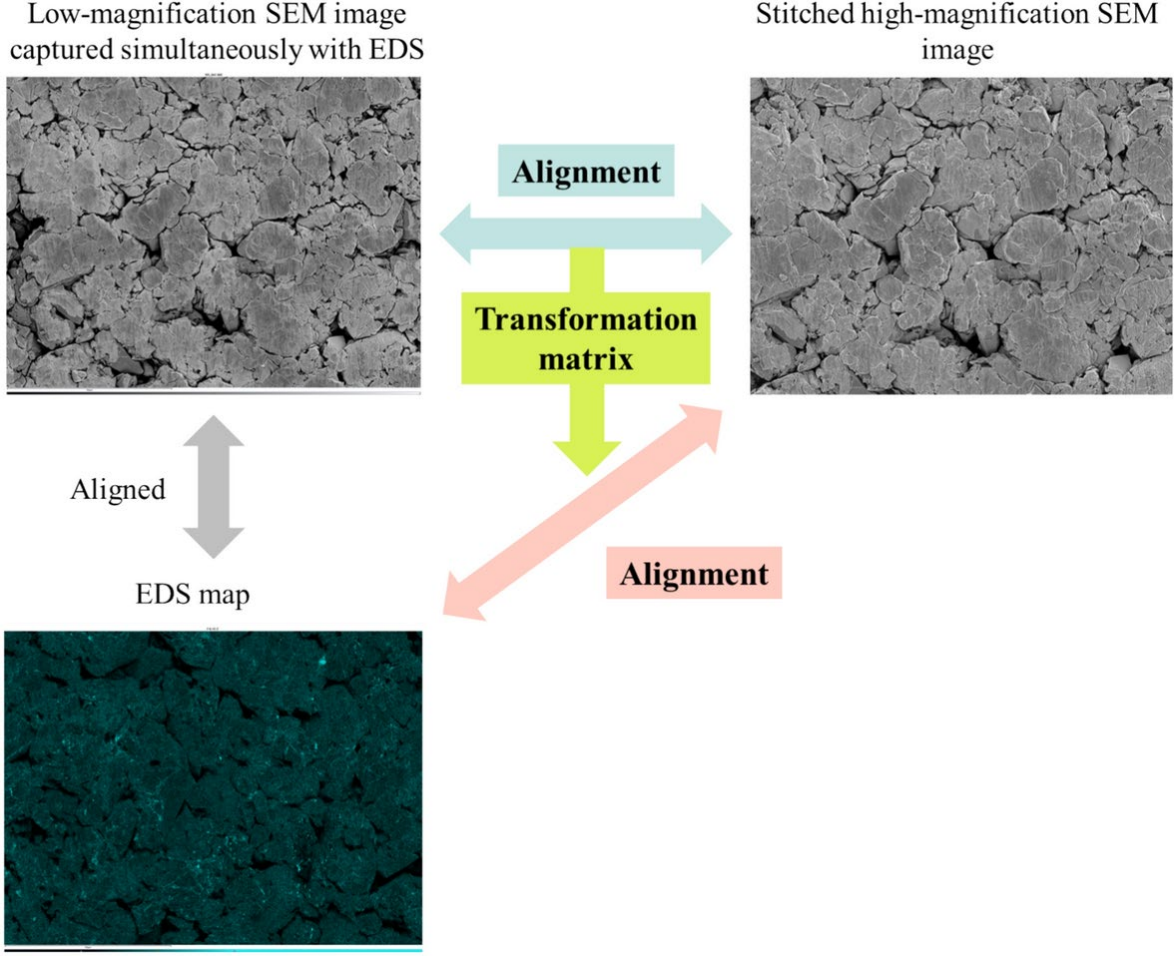
Alignment of the SEM images and EDS maps.

Integrated quantitative analysis combining the SEM images and EDS maps requires alignment of the images. Although the EDS map (Fig. 11, bottom left) and low-magnification SEM image taken simultaneously with the EDS measurements (Fig. 11, top left) are generally aligned, the stitched high-magnification SEM image (Fig. 11, top right) and low-magnification SEM image are not aligned. Therefore, the stitched high-magnification SEM image and EDS map are also not aligned. In this study, we calculated the transformation matrix aligning the low-magnification SEM image taken simultaneously with the EDS measurement with the stitched high-magnification SEM image and used it to align the EDS map with the stitched high-magnification SEM image. The stitching of the SEM images prior to alignment serves two purposes: First, the EDS maps are acquired at low magnification, whereas the SEM images are captured at high magnification. Therefore, by stitching the SEM images, their field of view matches that of the EDS maps, enabling more accurate alignment. Second, performing alignment after stitching allows the comparison of the global structural features, enhancing the precision of the alignment process. The specific alignment algorithm^39,45^ uses oriented FAST and rotated BRIEF (ORB) to detect image keypoints and calculate descriptors, followed by brute-force matching and similarity evaluation between descriptors based on the Hamming distance to determine the correspondences between images. The projection transformation matrix is then calculated from the correspondences selected by the random sample consensus (RANSAC) algorithm. The position alignment is completed by applying the projection transformation based on the transformation matrix.

#### Step A-5: patch division of SEM images and EDS maps

The aligned SEM images and EDS maps are divided into fixed-sized patches to create a dataset of patched SEM images and EDS maps for subsequent analysis. When training the machine learning models during the analysis, a randomly selected portion of the dataset was used as training data.

### Step B: microstructural analysis

#### Step B-1: feature extraction from SEM images

The features of the microstructures are extracted from each patched SEM image. In general, the methods for feature extraction from images can be categorized into those based on classical image features and those based on deep learning. Classical features include keypoint-based features such as scale-invariant feature transform (SIFT), speeded-up robust features (SURF), ORB^45^, as well as textural features^24,25^ such as local binary patterns^46^, and gray-level co-occurrence matrix (GLCM)^47–49^. Among these, the GLCM features, known for their high interpretability, quantify differences in human intuitive perception of images, such as “rough” or “smooth”^47,49–52^. Furthermore, the GLCM features have been applied in material image analysis and have been proven effective in microstructural analysis based on SEM images of steel^53–56^ and concrete^57^.

Recently, deep-learning-based image feature extraction models like convolutional neural networks (CNNs) and vision transformers (ViTs) have gained attention^39^. The effectiveness of the features extracted by machine learning models in material science image analysis has been demonstrated^58–62^, achieving high accuracy in tasks such as regression^63^, segmentation^64–66^, and super-resolution^67,68^. Although machine-learning-based models capture complex features that are difficult for the human eye to detect, their complexity makes intuitive interpretation challenging^60,69^.

The goal of this study was to develop an objective analytical process that provides consistent results with traditional intuitive insights rather than achieving high accuracy in a conventional sense (e.g., regression or classification accuracy); therefore, classical features were adopted. In particular, for SEM image analysis, it is important to capture not only keypoint features but also overall image features; hence, GLCM features were used for microstructure analysis. Following the method by Tsutsui et al*.*^56^, we used a total of 17 types of GLCM features: correlation, contrast, cluster prominence, cluster shade, dissimilarity, angular second moment (ASM), energy, entropy, homogeneity, maximum probability, inverse difference normalized, inverse difference moment normalized, sum average, sum entropy, sum variance, difference variance, and difference entropy. Among these, contrast, entropy, and homogeneity are particularly efficient for microstructural analysis. Contrast captures the sharpness of the boundaries between rough and smooth areas, emphasizing the transitions at the edges of the structural features. Entropy quantifies the complexity of the microstructure by measuring the randomness in pixel intensity distributions, making it an essential indicator for identifying highly irregular regions. Moreover, prior research by Tsutsui et al*.*^56^ identified homogeneity as an essential feature for distinguishing microstructures in SEM images of steel, suggesting its relevance in similar material analyses. For each of these 17 GLCM features, we varied the distance and angle parameters in N and M ways, respectively, and calculated a total of 17 × N × M dimensional features.

#### Step B-2: dimensionality reduction of SEM image features

Dimensionality reduction was performed to reduce the calculated redundancy of the SEM image features and to visualize the features on a two-dimensional plane. Standard methods for feature dimensionality reduction include principal component analysis (PCA)^70^, t-distributed stochastic neighbor embedding (t-SNE)^71^, and uniform manifold approximation and projection (UMAP)^72^. T-SNE and UMAP are known for their ability to place similar feature vectors of a high-dimensional space close to each other in a low-dimensional space. However, these model-free algorithms require the simultaneous input of all the features of the analytical target, rendering them unsuitable for dimensionality reduction of high-dimensional features of large datasets. Thus, in this study we used parametric UMAP^73^, which trains the dimensionality reduction model through a neural network to reduce the dimensionality of features to two dimensions. After training the dimensionality-reduction model using the features of the training data, the model was used to reduce the dimensionality of the features of the remaining data.

#### Step B-3: clustering of SEM image features

Clustering was performed to group the SEM patch images based on differences in microstructural features. While methods such as supervised classification and segmentation using manually annotated data could be considered for grouping the image features, we employed unsupervised clustering to ensure objective grouping. Clustering methods include k-means, the Gaussian mixture model (GMM), density-based spatial clustering of applications with noise (DBSCAN), and spectral clustering^74^. In this study, we used spectral clustering, which is known for its applicability to nonlinear data clustering, to cluster features after dimensionality reduction.

Because clustering results depend on the hyperparameters of the number of clusters, it is important to evaluate these results and determine the optimal number of clusters. For instance, too few clusters may result in overly simplistic and trivial clustering, whereas too many clusters can lead to complexities that are difficult for humans to interpret. Known methods for evaluating and optimizing clustering results include modifying the results based on an analyst’s expert knowledge through feedback^21,75^. However, to achieve an objective analysis that does not rely on an analyst’s subjectivity, quantitative evaluation metrics were used in this study to determine the number of clusters. Quantitative metrics for evaluating clustering results include the silhouette coefficient^76^ and the Calinski-Harabasz (CH) criterion^77^, which consider the cohesion within clusters and the separation between clusters. In this study, the clustering result that maximizes the CH criterion was adopted; spectral clustering was performed multiple times on the training data with varying numbers of clusters and the number of clusters for which the CH criterion is maximized was selected.

Notably, similar to dimensionality reduction, spectral clustering is a model-free algorithm, unsuitable for processing large datasets. Therefore, a predictive model for clusters was trained using the low-dimensional features of the training data and the adopted clustering results. Any classification model can be adopted for the predictive model; in this study, we used the simplest nearest-neighbor model^70^. The trained model was then used to cluster the low-dimensional features of the remaining data.

### Step C: elemental distribution analysis

#### Step C-1: calculation of total elemental distribution

The elemental distributions across the entire dataset of the EDS maps were calculated. The brightness values of each patched EDS map were averaged, and the frequency distribution of the average brightness values represented the overall elemental distribution.

#### Step C-2: calculation of elemental distribution by cluster

To analyze the differences in elemental distribution for areas with different microstructures, the elemental distribution of each cluster obtained through microstructural analysis was calculated. Specifically, patched EDS map groups corresponding to each cluster (patched EDS maps at the same locations where the SEM image cluster corresponded to the target cluster) were extracted from the patched EDS map dataset, and a frequency distribution of the average brightness values was created.

#### Step C-3: calculation of similarity between elemental distributions

To quantify the contribution of the elemental distribution by cluster to the overall elemental distribution, the similarity between these distributions was calculated. The Kullback–Leibler (KL) divergence^70^ and Wasserstein distance^26^ are quantitative metrics for comparing distributions. However, because the KL divergence does not satisfy mathematical properties, such as symmetry and triangle inequality, it is not suitable for intuitive interpretation of distance. Therefore, the similarity between the elemental distributions was quantified by taking the inverse of the Wasserstein distance between the elemental distributions.

### Lithium-ion battery electrode samples

The proposed method was applied for the analysis of lithium-ion battery electrode samples, using data obtained from three commercially available lithium-ion batteries (Panasonic NCR18650B) with varying degrees of degradation (capacity retention rates). Two batteries were degraded (capacity reduced) during high-temperature storage. The third battery was not subjected to high-temperature storage and served as the new battery. High-temperature storage was conducted at a state of charge (SOC) of 100% and a voltage of 4.2 V, at a storage temperature of 60 °C. The storage duration of the degraded batteries varied, resulting in different capacity retention rates (Table 1).

**Table 1:** Degree of degradation of the tested lithium-ion batteries.

Storage duration [days]	Post-storage capacity [mAh]	Initial capacity [mAh]	Capacity retention rate [%]
–	–	3390.4	100
105	3226.9	3380.3	95.5
387	2876.3	3375.9	85.2

**Fig. 12:**
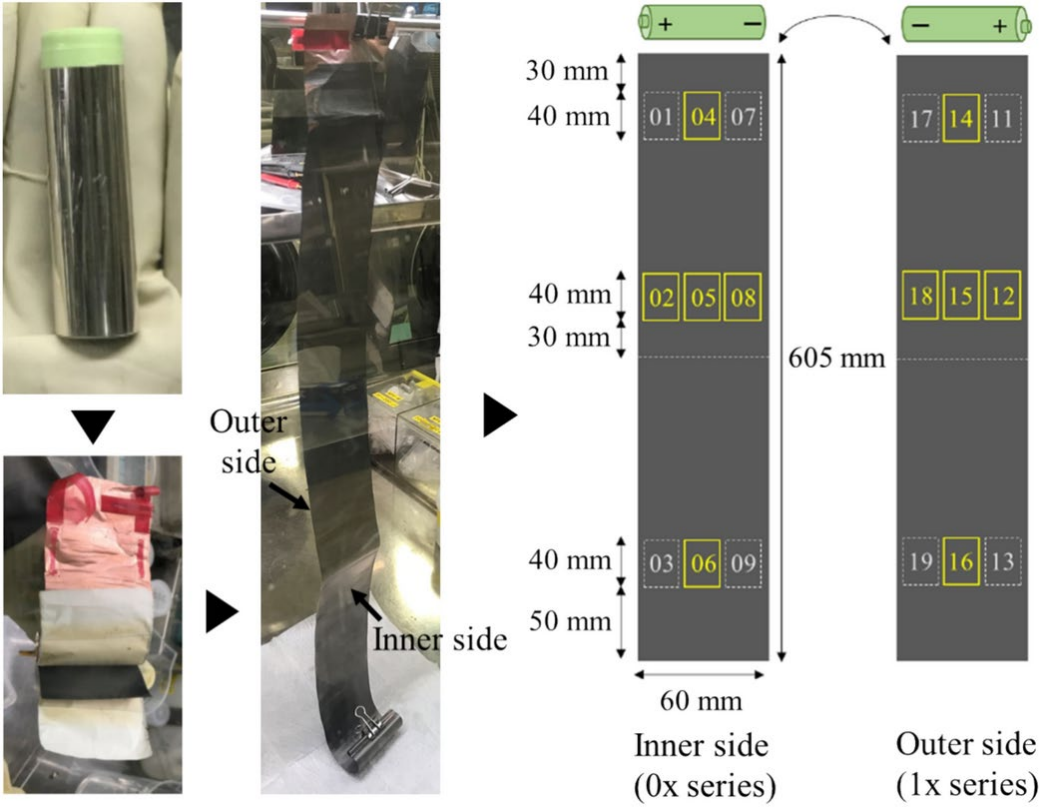
Sampling locations on the lithium-ion battery electrode. The ten locations enclosed in yellow frames represent the sampling sites.

The capacity of each battery was measured by fully discharging the batteries through a constant current–constant voltage (CC–CV) mode after CC–CV charging. As shown in Table 1, the longer the storage duration, the lower the capacity retention rate. After capacity measurement, each battery was fully discharged and disassembled, and the positive and negative electrodes were separated. The graphite negative electrodes were washed with dimethyl carbonate to remove the electrolyte, followed by drying. Ten samples were taken from each electrode to cover different areas evenly, including regions near the edge and the center of the electrode. The sampling locations are shown as yellow frames in Fig. 12. This approach ensures that the dataset represents the full structure of the electrode for SEM–EDS measurements. The aforementioned procedures were conducted inside an argon-filled glove box to prevent air exposure of the samples. Three levels of degradation conditions were applied involving ten samples each, thus affording a total of 30 samples.

### SEM–EDS measurement settings

The measurement conditions for the SEM images and EDS maps obtained in Step A were as follows: During the automated capture process, high-magnification SEM images were sequentially captured in a 10 × 10 grid pattern with a 10% overlap rate for each battery (ten samples per battery). A total of 3000 SEM images were therefore captured across all samples. SEM imaging was performed using a Hitachi High-Tech SU7000 for backscattered electron imaging, with the conditions set to a magnification of 10 k times, an acceleration voltage of 2 kV, and an image size of 1280 × 960 pixels. Subsequently, one EDS map and one low-magnification SEM image covering the area of the high-magnification SEM image group were measured. EDS mapping was performed using an Oxford Instruments Ultim Max 170 for the F, O, and C maps set to a magnification of 1 k times, an acceleration voltage of 2 kV, and an image size of 2048 × 1536 pixels. The low-magnification SEM images were captured under identical conditions.

### Image processing conditions

In Step A, the SEM images and EDS maps were stitched, aligned, and then divided into 64 × 64 pixel patches (635 × 635 nm), affording 436,800 pairs of patched SEM images and EDS maps. In Step B, two distance parameters (1 and 10) and four angle parameters (0, π/4, π/2, and 3π/4) were used for calculating the 17 types of GLCM features for each SEM patch image, producing 136-dimensional features. The numbers of clusters considered for spectral clustering were set to six options: 3, 4, 5, 6, 7, and 8. To train the dimensionality reduction and clustering models, 50,000 of the 436,800 SEM patch images were randomly selected.

## Conclusion

In this study, we proposed an objective SEM–EDS analytical process that addresses the limitations of conventional SEM–EDS analysis, namely the lack of comprehensiveness and quantitativeness. By combining the automated capture of large-scale SEM datasets with the quantitative analysis of microstructures and elemental distributions, this method offers a robust and unbiased evaluation of the relationship between surface structure and composition. Degradation analysis of lithium-ion battery electrodes revealed that the proposed method yields results consistent with traditional subjective outcomes, while providing additional quantitative clarity. This analytical approach has broad applicability beyond the specific case of lithium-ion battery electrodes, as it establishes a general framework for integrating microstructural and compositional data. This is particularly valuable for new materials with limited prior analytical information, as the method reduces reliance on expert subjectivity and enhances reproducibility.

Future work on SEM–EDS analysis should focus on enhancing the efficiency of data collection and improving the interpretability of the features. Although SEM image capturing is an automated procedure, EDS measurements are still performed manually. For SEM image-based surface structural analysis, capturing fine details at high magnification is essential, whereas for compositional analysis using EDS maps, lower magnification maps are deemed sufficient for analyzing elemental distribution. However, the automation of EDS measurements is essential when analyzing large datasets. Furthermore, our approach involves transforming 136- dimensional GLCM features into two-dimensional abstract features using parametric UMAP to visualize features on a two-dimensional plane and facilitate interpretation. However, this process complicates the direct comparison of individual feature contributions to the clustering results. Therefore, improving the interpretability of GLCM features through methods like local interpretable model-agnostic explanations (LIME)^78^ or Shapley additive explanations (SHAP)^79^ will be a significant task moving forward.

Additionally, optimizing the selection of analytical methods remains an important topic for improving practical applicability. While this study demonstrated the effectiveness of GLCM-based features in explaining traditional qualitative results, a systematic evaluation of the different methods, including deep learning-based features, could be beneficial for addressing more complex problems. However, deep learning features generally lack interpretability, which can make them less suitable for applications requiring reliable decision-making. Balancing the flexibility of deep learning with the interpretability of classical features, possibly through advanced interpretability techniques (e.g., LIME, SHAP), represents a promising direction for future research. Furthermore, quantitative evaluation metrics, such as the separability of feature distributions or elemental maps among clusters, could provide objective criteria for comparing analytical methods. These efforts would contribute to a more robust and generalizable framework, although they extend beyond the scope of this paper.

## Supplementary Information

Supplementary Information.

## Data Availability

The datasets generated and analyzed in the current study are not publicly available but are available from the corresponding author upon reasonable request.

## Code availability

The underlying code for this study and the datasets are not publicly available. However, they are available to qualified researchers upon reasonable requests from the corresponding author.
