# Supplementary material for: A comprehensive and quantitative SEM–EDS analytical process applied to lithium-ion battery electrodes
Source: Sci Rep. 2025 Feb 13;15:5428. doi: 10.1038/s41598-025-89362-w (PMC11825835; doi:10.1038/s41598-025-89362-w)
Supplement: Supplementary file 1 — Supplementary Information. [file 41598_2025_89362_MOESM1_ESM.docx]

**Supplementary Information**

The supplementary details regarding the number of clusters are as follows: Clustering was performed multiple times on the training data with varying numbers of clusters and the Calinski-Harabasz (CH) criteria are presented in Supplementary Table S1.

Supplementary Table S1. Calinski-Harabasz criteria for each clustering result.

| Number of Clusters | CH Criterion |
| --- | --- |
| 3 | 50817 |
| 4 | 47218 |
| 5 | 44371 |
| 6 | 47038 |
| 7 | 48709 |
| 8 | 47194 |

Based on Supplementary Table S1, the clustering result with the highest CH criterion i.e. three clusters, was adopted.
